# Assessment of Egyptian Mothers’ Knowledge and Domestic Management Practices of Fever in Preschool Children in Zagazig City, Sharkia Governorate

**DOI:** 10.3390/children9030349

**Published:** 2022-03-03

**Authors:** Eman H. Waly, Huny M. Bakry

**Affiliations:** Community, Environmental and Occupational Medicine Department, Faculty of Medicine, Zagazig University, Zagazig 44519, Egypt; honeybakry@gmail.com

**Keywords:** fever, mothers, knowledge, practice, preschool, children

## Abstract

(1) Background: Childhood fever is a frequent reason for health care visits. Parents are worried about fever and its complications and show variation between their knowledge about managing fever and real practice, which are affected by many factors and beliefs. This study aimed to assess knowledge of Egyptian mothers about fever of preschool children and its domestic management and the relation between them and to identify sociodemographic factors affecting mothers’ knowledge and practice. (2) Methods: a cross-sectional study was conducted at the pediatric outpatient clinic at Zagazig University Hospitals among 297 mothers with preschool children. A structured questionnaire consisting of three parts assessed the sociodemographic characteristics, mothers’ knowledge about childhood fever, and its management practices. (3) Results: 37.7% of mothers had good knowledge about childhood fever, and 23.9% showed good management practices. Young mothers, less number of children, high education, sufficient income, and good knowledge were the significant predictors of domestic management practices towards childhood fever. (4) Conclusions: The Egyptian mothers showed insufficient levels of knowledge and domestic management practices towards preschool childhood fever. Health education interventions should be targeted to mothers to improve their knowledge and practice.

## 1. Introduction

Fever is an increased temperature above the normal values for an individual to be 38 °C or greater due to the rise in the body’s temperature hypothalamic set-point [1,2]. Fever itself is not considered an illness. Fever is a part of the human body’s controlled physiological response to infections and inflammatory and immunological disorders [3]. With fever, body temperature is regulated by the hypothalamic center balancing heat production and loss not to exceed the upper limit of 41 °C [4].

Fever is a frequent symptom of various diseases affecting preschool children, and it is one of the most common causes of the parents’ anxiety and seeking health care advice. It is the most frequent non-traumatic cause for pediatric emergency visits [4,5]. A study was conducted in Egypt revealed that childhood fever represented 15.5% of all the complaints attending the pediatric emergency services at El-Behera Hospitals [6]. The African rural areas recorded high incidences of fever in various countries [7]. Moreover, it represented more than 50% of visits to pediatric outpatient clinics in several Sub-Saharan African countries [8].

The misunderstanding of parents to childhood fever’s proper definition, causes, and management contribute to aggravated parental fear that is known as fever phobia. Fever phobia leads to rigorous attempts to lower the child’s temperature to be normothermic, such as immediate giving of antipyretics without medical prescription [9,10]. This behavior is also due to their extreme fear of complications of fever as febrile convulsions, brain damage, or death [9,10].

Knowledge is known as a set of information that individuals need to administer and manage their health condition [11]. Knowledge combined with parents’ decision-making, values, perceived barriers, motivation, and social beliefs, can lead to positive practices towards the management of diseases [12]. Parents’ decisions about medication and healthcare-seeking behavior in acute illness are influenced by knowledge, sociodemographic characters, and public beliefs [13]. Previous studies in different parts of the world revealed varied parents’ knowledge and practices toward childhood fever management [13,14,15,16,17]. Therefore, it is essential to assess the parental knowledge and behavior towards their feverish child and their relation to parental sociodemographic characters to avoid mismanaging of fever at home and to decrease the overload on healthcare services.

This study aims to assess the knowledge and domestic management practices of Egyptian mothers towards preschool childhood fever and the relation between them and to identify some sociodemographic factors that are related to the Egyptian mothers’ knowledge and management practices towards preschool childhood fever.

## 2. Materials and Methods

### 2.1. Study Design and Setting

A cross-sectional study was conducted at the general pediatric outpatient clinic at Zagazig University Hospital over two months (July–August 2021).

### 2.2. Sample Size and Sampling

A sample of 297 mothers of preschool children was drawn from a total population of 6670 based on the prevalence of correct knowledge among mothers regarding domestic management of fever (28%), which was calculated from the pilot study and confidence interval 95%. A pilot study was conducted before data collection on 20 mothers to determine the prevalence of the factor under study and to check the clarity and applicability of the questionnaire, and necessary modifications were made accordingly. The participants who were included in the pilot study were excluded from the final results. Participants were selected by systematic random sample, which is a probability sampling based on a random selection of the first participant with fixed periodic intervals.

### 2.3. Inclusion Criteria

Mothers who have attended the pediatric outpatient clinics with feverish children aged five and less.

### 2.4. Data Collection Tool

A structured questionnaire was designed to assess the knowledge and domestic management practices of the mothers towards the fever of preschool children. The structured questionnaire encompassed three sections, gathering information about mothers’ sociodemographic characteristics, knowledge about childhood fever, and its management practices, respectively. The first section about sociodemographic data included age, number of children, education level, working status, and income. The second section was to assess the knowledge of the mothers about fever and its management through 8 multiple-choice (single correct answer) questions, including the definition of fever (≥38 °C) [1]; the best site for measuring the temperature of under 5-year-old children (rectal for the children of ≥3 years and oral or axillary for 4–5 years old children) [18]; severe complications of fever (loss of consciousness, convulsions, brain damage, and death) [9,10]; types of antipyretics (Acetaminophen and Ibuprofen) [19]; choosing the proper type of antipyretic (Acetaminophen and Ibuprofen combination or alternating treatment regimen) [20]; that an alternating treatment regimen is more effective in managing fever (more effective and the best method) [20]; in case of high fever, increasing the antipyretic dose is beneficial or not (not beneficial) [19]; and that the antipyretic dose calculation is dependent on (child’s weight) [21]. The correct answers that were mentioned before between brackets next to each item were scored 2, and the incorrect or do not know answers were scored 0 with a total score of 16. The third section was designed to assess the practices of the mothers to manage the fever of their children through 7 multiple-choice (single correct answer) questions, including the method of fever diagnosis (thermometer), the site used for measuring temperature by using a thermometer on under-five children (rectal, oral, or axillary), what to do once the fever is detected (visiting a physician or health care facility), calculation of antipyretic dose (according to child’s weight), using appropriate way to administer the prescribed doses (graduated syringe) [19], using alternative medicine (lukewarm water compress, or drinking fluids) [19], and administration of antibiotics after medical prescription (yes). The correct answers that were mentioned before between brackets next to each item were scored 2, and the incorrect answers were scored 0 with a total score of 14. Knowledge and practices of fever management were considered bad if (≤50%) of the total score, fair or moderate if (>50%–75%) of the total score, and good if (>75%) of the total score [22]. The validity of the questionnaire was determined by consulting 5 faculty members who are experts in the field [23,24,25]. The questionnaire validity index/average was 1, which was based on the calculation of the content validity of each item separately, and then the average was calculated. All experts agreed on the validity of all items and no modifications. However, minor modifications were performed after the pilot study in the Arabic language of two questions to be more understandable, and the experts did not mind the modifications.

### 2.5. Statistical Analysis

After data collection, data were coded manually based on the scoring system that was explained before. Then, data were entered and analyzed using a statistical package of social sciences SPSS version 24 [26]. Descriptive analysis was performed, and data were presented as numbers and percentages in descriptive tables and figures. Spearman’s correlation test was used to measure the correlation between mothers’ knowledge and management practice towards preschool children’s fever and some sociodemographic factors and to measure the correlation between the knowledge of mothers about fever and their management practices. The *p*-value was considered to be significant at ≤0.05. Ordinal regression analysis was carried out to determine the independent predictors of proper management practices for childhood fever.

### 2.6. Ethical Considerations

Data collection started after getting approval from Zagazig University Institutional Review Board (IRB). Official permissions from the heads of the pediatrics outpatient clinic and the department were obtained before starting data collection. The purpose of the study was stated on the cover page of the questionnaire. Before data collection, verbal consent was taken from mothers, their identities were kept anonymous, and they were guaranteed the confidentiality of their data and that it will be used for the research only.

## 3. Results

### 3.1. Sociodemographic Characteristics

The total number of mothers included in this study is 297. 215 (72.4%) of them have more than three children. The majority of mothers have neither job nor sufficient income that suffices the basic needs of life, such as food and clothing. One hundred and twelve (37.7%) of all mothers have good knowledge about preschool childhood fever, and 71 (23.9%) of them show good management practices toward childhood fever (Table 1).

### 3.2. Knowledge and Practices of Mothers towards Preschool Childhood Fever and Its Management

**Figure 1:**
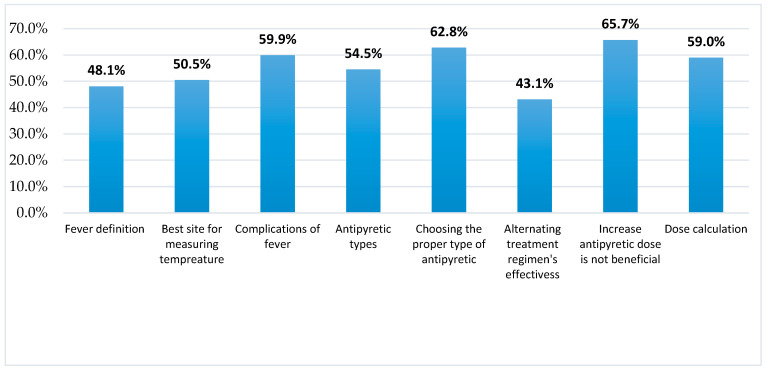
Percentages of correct knowledge scores of mothers about preschool childhood fever and its management.

Figure 1 shows that approximately half of all mothers responded correctly regarding the definition of fever (48.1%), best site for measuring the temperature of under 5-year children (50.5%), and types of antipyretics (54.5%). Better responses are observed regarding the complications of fever (59.9%), choosing the proper type of antipyretic (62.8%), increasing the antipyretic dose is not beneficial in lowering high fever (65.7%), and dose calculation of antipyretic (59%).

**Figure 2:**
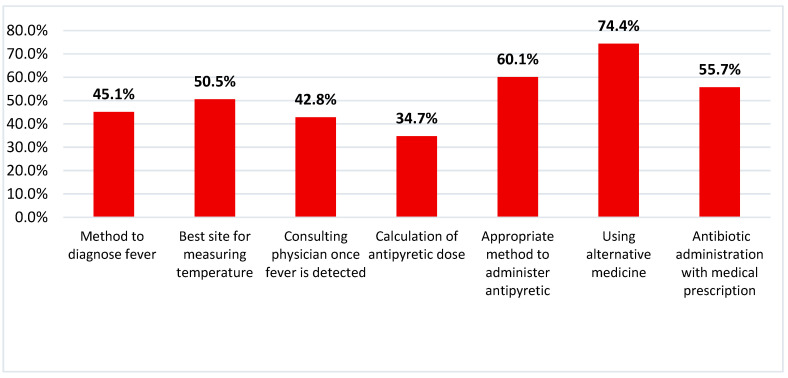
Percentages of correct management practices scores of mothers toward preschool childhood fever.

Mothers show correct responses regarding diagnosing fever by forehead touch or thermometer (45.1%), consulting a physician or health care facility once the fever is detected (42.8%), and the proper site for measuring temperature by thermometer for under-five children (50.5%). Only 34.7% of the studied group can correctly calculate the dose of antipyretic. Better responses are observed regarding the proper way to administer the prescribed antipyretic doses, using alternative medicine such as lukewarm water compresses, and administration of antibiotics with medical advice (Figure 2).

### 3.3. Relation between Sociodemographic Characteristics and Knowledge and Management Practices

Table 2 shows that the mothers’ knowledge about childhood fever is significantly correlated with younger mothers and a lesser number of children. Moreover, the knowledge is significantly correlated with higher educated mothers, working mothers, and sufficient income. The mothers’ practices for the management of fever are significantly correlated with younger mothers, lesser number of children, higher educated mothers, and with sufficient income.

Finally, the ordinal logistic regression analysis revealed that young aged mothers, less number of children, high educational level, sufficient income, and good knowledge about childhood fever were the significant independent predictors of proper (good) management practices towards childhood fever (Table 3).

## 4. Discussion

A widespread range of childhood diseases are accompanied by fever; most of them are managed at home before visiting a nearby health care facility. Misconceptions and beliefs regarding childhood fever were aroused in previous studies; thus, it shapes knowledge and practice of childhood fever management [13,15]. Traditional infant care practices that are deeply rooted in the rural communities are other issues that were aroused in a study conducted in Egypt [27]. Skarkia governorate is the third populous governorate in Egypt. Rural residents in the Sharkia governorate represent almost 77% of Sharkia governorate residents [28], and they are mainly served by Zagazig University hospitals. Therefore, the objectives of this study were to assess mothers’ knowledge and domestic management practices of fever among preschool children, as well as identify some factors that are related to their knowledge and management practices.

This study was conducted upon 297 Egyptian mothers of preschool children. Only 10.1% of mothers in the present study had university graduates, which is considered in Egypt as a high educational level. The education system in Egypt has three levels: basic education level for 4–14 years old (pre-primary, primary, and preparatory), Secondary education level for 15–17 years old, and high education level (university education) [29].

Regarding the knowledge of mothers towards childhood fever, our findings revealed that a third of the participants had good knowledge with a mean knowledge score (9.6 ± 2.9). Additionally, almost a quarter of mothers showed good practices for managing the fever of their children with a mean practice score (8.1 ± 2.8). A previous study that was conducted in France reported that 88.3% of parents with one or more children aged <5 years showed good knowledge about fever while only 53.6% of them showed good practice towards fever management [13]. Another previous study demonstrated that parents had good levels of general knowledge regarding fever and its management but showed a reluctant attitude or improper delayed action in managing fever. This improper attitude was assumed to be due to the lack of confidence of the parents to manage their children’s fever properly [14]. However, these findings were inconsistent with the results of a study in Saudi Arabia that revealed that (94.2%) of Saudi parents displayed poor knowledge about preschool childhood fever and 56.8% of them displayed poor management practices’ scores [15]. In the previous studies, the differences in the cultural backgrounds play an important role in the variability between their findings.

In the current study, approximately half of the mothers correctly defined fever as an increase in the body temperature to be ≥38 °C. An American study reported that 55% of parents had good knowledge about the correct definition of fever as (≥38 °C) [30]. Other studies reported better knowledge scores regarding fever’s definitions that were 83.8%, 67%, and 75% of all participants [8,15,31]. Moreover, half of the mothers in the current study correctly reported the best site for measuring the temperature of under 5-year children as rectal for children of ≥3 years and oral or axillary for 4–5 years old children. These findings were consistent with previous studies [8,15,31]. Previous studies revealed correct knowledge of parents about proper antipyretics, which are Acetaminophen alternating with Ibuprofen as a combination treatment regimen with the following percentages 91.2% and 71% [15,32] However, only half of the mothers in the present study reported correct knowledge. A possible explanation for these findings is that most of the mothers in the present study were not highly educated, and according to other studies that were conducted in Jordan and Kuwait, the level of education of parents is a determinant of their knowledge about childhood fever and its management [16,17].

In the present study, better responses were reported regarding the mothers’ knowledge about the complications of fever, choosing the proper type of antipyretics, increasing the dose of antipyretic is not beneficial and not recommended in managing pediatric fever, and the dose calculation of antipyretic is according to the child’s weight. These results were concordant with the findings of other studies that assessed the knowledge of the parents about the previous items [15,33].

Almost half of the mothers in the present study diagnosed fever properly by the thermometer and the rest by forehead touch. The results of previous studies revealed better responses regarding the proper method to diagnose childhood fever. Seventy-five percent and 87.8% of mothers in these studies used thermometers to diagnose their children’s fever [8,15]. Approximately half of the mothers in the present study consulted a physician or visited a health care facility once the fever was detected, which is less than the results reported in other studies [32,34]. This finding may be explained by the fact that the majority of the mothers in the present study had insufficient income. Therefore, they may prefer to take advice from relatives and neighbors for managing the fever to save the cost of visiting a physician or health care facility. This assumption is supported by an American study that linked the delayed visiting of health care facilities to low income [35]. The antibiotics should only be used with medical prescription as the irrational consumption of antibiotics can lead to the development of drug resistance and antagonistic reactions [8,36]. Half of the mothers in the current study used antibiotics without medical prescription. This finding was in agreement with the results of another study stating that 40.1% of the parents used antibiotics in managing pediatric fever without medical prescription [31].

Previous studies revealed that the sociodemographic variables such as age, the number of children, education, occupation, and family income had significantly affected the parents’ knowledge about childhood fever [16,17,37]. The previous finding was concordant with ours, revealing that the mothers’ knowledge about childhood fever and management practices were significantly correlated to some sociodemographic factors. The young mothers and mothers with fewer children had better knowledge and management practices for childhood fever. These findings may be explained by the fact that young mothers were better educated than older ones in this study, and as was mentioned before, the level of education is a main determinant of knowledge. In addition, the mothers with fewer children may be more anxious about their child’s illness, and once the fever is detected, they immediately seek medical care. On the other hand, two previous studies were reported that parents’ knowledge and management practices towards childhood fever were significantly positively correlated with parents’ age and the number of children that was explained by having better experience [15,16]. Moreover, the higher educated mothers and sufficient income showed better knowledge and management practices. A previous study that was conducted in Iran assessed the knowledge and performance of parents towards their childhood fever was consistent with these findings. The Iranian study reported that the parents’ education raises their knowledge about pediatric fever and its management. In addition, better education decreases the parents’ phobia from fever and its complications [38].

The present study exhibited a positive correlation between mothers’ knowledge about preschool childhood fever and the proper management practices towards fever which were in agreement with the results of another study that found a positive correlation between knowledge of mothers about fever and their practices in managing fever [39].

In the current study, the results of the ordinal regression support the suggestion that age, number of children, educational level, family income, and good knowledge about childhood fever and its management are the most important factors predicting the proper management practices towards preschool childhood fever.

The findings of the current study suggest emphasizing the importance of doctor–patient relationships in addition to the introduction of health education campaigns in Sharkia governorate to guide the mothers to the proper domestic management practices towards childhood fever and the importance of proper health-seeking behavior.

## 5. Conclusions and Recommendation

This study indicates more than one-third of mothers had good knowledge about childhood fever, and a quarter of them showed good management practices towards preschool childhood fever. The mothers’ management practices towards fever were significantly related to some factors, which are young age, fewer children, higher educational level, sufficient income, and good knowledge.

According to the results of this study, we recommend the introduction of health education interventions to target parents to be fully informed and empowered caregivers for their feverish children. Similar population-based studies are advised to achieve the generalization of results. Moreover, further studies are recommended to assess other factors that may be related to the domestic management practices towards childhood fever.

## 6. Limitation of the Study

The results of this study cannot be generalized to the varied and large Egyptian population as the data of the current study were collected from regional hospital settings.

## Figures and Tables

**Table 1:** Sociodemographic characteristics of mothers and their knowledge and management practices of preschool childhood fever.

Items	No. (Total = 297) (%)
Age	
≤30 Years	142 (47.8)
>30 Years	155 (52.2)
Number of Children	
<3	82 (27.6)
≥3	215 (72.4)
Educational level	
Preparatory and Secondary education	267 (89.9)
University graduate education	30 (10.1)
Working status	
Not working	274 (92.3)
Working	23 (7.7)
Income	
insufficient	237 (79.8)
Sufficient	60 (20.2)
Knowledge about preschool childhood fever	
Bad	139 (46.8)
Moderate	46 (15.5)
Good	112 (37.7)
Practice towards preschool childhood fever management	71 (23.9)
Bad	
Moderate	83 (27.9)
Good	143 (48.1)

**Table 2:** Correlation between sociodemographic characteristics and mothers’ knowledge and management practices of fever, and correlation between mothers’ knowledge and practice.

Items	Knowledge		Practice	
	*r* *	*p*-Value	*r* *	*p*-Value
Age	−0.137	0.018	−0.354	<0.001
Number of Children	−0.238	<0.001	−0.311	<0.001
Educational Level	0.363	<0.001	0.210	<0.001
Working Status	0.270	<0.001	0.006	0.913
Income	0.221	<0.001	0.172	0.003
Knowledge	1		0.490	<0.001

*r* * = Spearman’s correlation coefficient.

**Table 3:** Ordinal logistic regression analysis of predictor variables on mothers’ management practices of fever.

Items	Estimate	Std. Error	Wald	df	Sig.	95% C.I.	
						Lower Bound	Upper Bound
Age (>30 years old)	−1.039	0.310	11.258	1	<0.001	−1.646	−0.432
Number of Children (≥3)	−1.323	0.416	10.097	1	<0.001	−2.139	−0.507
Educational Level (University graduate)	1.533	0.375	16.700	1	<0.001	2.269	0.798
Working Status (Working)	−0.190	0.326	0.340	1	0.560	−0.829	0.449
Income (Sufficient)	1.687	0.502	11.288	1	<0.001	0.703	2.672
Knowledge (Good)	1.147	0.171	45.015	1	<0.001	0.812	1.481

The reference of the dependent variable is good management practices towards child’s fever.

## Data Availability

The data of this study is available on request from the corresponding author after getting approval from the local Research Ethics Board.
